# Prognostic factors for visual acuity in patients with Leber's hereditary optic neuropathy after rAAV2‐ND4 gene therapy

**DOI:** 10.1111/ceo.13515

**Published:** 2019-05-08

**Authors:** Yong Zhang, Xin Li, Jiajia Yuan, Zhen Tian, Hongli Liu, Dan Wang, Bin Li

**Affiliations:** ^1^ Department of Ophthalmology Taihe Hospital (Affiliated Hospital of Hubei University of Medicine) Shiyan China; ^2^ Department of Ophthalmology, Tongji Hospital Huazhong University of Science and Technology Wuhan China

**Keywords:** Leber's hereditary optic neuropathy, gene therapy, ND4, prognosis

## Abstract

**Importance:**

Factors affecting visual acuity prognosis after gene therapy in Leber's hereditary optic neuropathy (LHON) patients with mutation at site 11 778 are unknown.

**Background:**

To analyse correlations between visual acuity prognosis and baseline characteristics of LHON after rAAV2‐ND4 gene therapy.

**Design:**

Retrospective study.

**Participants:**

Fifty‐three LHON patients with a mutation at site 11 778.

**Methods:**

Single‐eye intravitreal injection of rAAV2‐ND4.

**Main Outcome Measures:**

Sex, onset age, duration of disease, best‐corrected visual acuity (BCVA), visual field index (VFI) and mean deviation (MD) were recorded for all patients at baseline. BCVA was recorded at 1‐ and 3‐month follow‐up visits after gene therapy. Correlations between BCVA prognosis and baseline characteristics were analysed by univariate analysis. Logistic regression analysis was performed on independent factors affecting BCVA prognosis.

**Results:**

Univariate analysis showed significant differences in the VFI and MD of the injected eye between BCVA improvement and non‐improvement groups after 3 months of treatment, with greater VFI and smaller absolute MD in the BCVA improvement group. Logistic regression showed that VFI and baseline BCVA were independent prognostic factors for visual acuity. The correlation between VFI and MD was statistically significant.

**Conclusions and Relevance:**

VFI and baseline BCVA were correlated with the visual acuity prognosis of LHON patients receiving gene therapy, with greater baseline VFI and better baseline BCVA predicting better visual acuity prognosis. MD was strongly correlated with VFI and might be correlated with gene therapy prognosis. This finding may form a basis for predicting the efficacy of gene therapy in these patients and guiding subsequent treatment.

## 1 INTRODUCTION

Leber's hereditary optic neuropathy (LHON) is a maternally inherited ophthalmopathy caused by mitochondrial DNA mutations. In China, the most common primary mutation site of LHON is 11778G>A,^1^ which is also associated with the worst visual acuity prognosis^2^; most affected patients have visual acuity below 0.1.^3^ According to reports from China and other countries, the proportion of 11 778 mutations is between 55.5% and 90.9%.^1^, ^4^, ^5^ Gene therapy is currently the most promising treatment method for this disease, and its safety and efficacy have been well‐demonstrated.^6^, ^7^ The results of an early‐stage clinical trial (ClinicalTrials.gov Register No.: NCT01267422) showed significant efficacy, with more than 66% of patients achieving significant improvement in visual function.^8^, ^9^, ^10^ Thus far, gene therapy is effective in some patients and ineffective in others. Therefore, it is important to identify the factors affecting visual acuity prognosis in LHON patients with mutation at site 11 778, after receiving gene therapy. In an analysis of data from patients who exhibited spontaneous recovery of visual acuity, differences in baseline characteristics were found to affect the prognosis of LHON patients.^3^ Therefore, this study investigated LHON patients with mutation at site 11 778 treated with rAAV2‐ND4 gene therapy and analysed correlations between visual acuity prognosis and baseline characteristics of patients treated with gene therapy.

## 2 METHODS

### 2.1 Study subjects

A retrospective analysis was performed using data from 53 LHON patients with a definite diagnosis of mutation at site 11 778, confirmed by genetic testing; all patients were treated in Tongji Hospital of Huazhong University of Science and Technology during the period from December 2017 to April 2018. All patients received single‐eye intravitreal injection of rAAV2‐ND4 (0.05 mL) as gene therapy (ClinicalTrials.gov Registered No: NCT03153293). Inclusion criteria: All patients had normal general check‐up results and no other abnormalities on ocular examination except for optic neuropathy. Exclusion criteria: Systemic and ocular diseases that affected the patient's visual function, use of other drugs within the prior 6 months that may affect the accuracy of gene therapy (such as idebenone, vitamins or traditional Chinese medicine), smokers and heavy drinkers, postoperative complications that affected the patient's visual function, incomplete data or less than 3 months of follow‐up. The injected eyes of all patients were enrolled as the study objects.

### 2.2 Study methods

#### 2.2.1 Basic information

Patient sex, age at onset and duration of disease were collected and recorded.

#### 2.2.2 Eye examination

The best‐corrected visual acuity (BCVA) was measured by a 2.5‐m standard logMAR visual acuity scale (Wenzhou Xingkang Medical Science Technology Co., Ltd.). The data were checked and recorded by the same person. On the basis of BCVA changes, patients were divided into the improvement and non‐improvement groups, with BCVA improvement ≥0.3 logMAR defined as improvement^11^, ^12^, ^13^ and BCVA improvement <0.3 logMAR as non‐improvement or reduction. Visual field examination was performed using the Humphrey visual field analyser (Carl Zeiss 740i, Germany); the test procedure was performed using the central 30‐2 threshold test and SITA fast algorithm, primarily collecting parameters of visual field indices (VFIs) and mean deviation (MD).

#### 2.2.3 Choice of injected eye

The eye with poorer visual acuity was selected for intravitreal injection of rAAV2‐ND4. If the visual acuities of the two eyes were the same, the right eye was chosen for intravitreal injection.

### 2.3 Statistical analysis

Data analysis was performed using spss 22.0 statistical software. Continuous variables were expressed as mean ± SD, and categorical variables were expressed as n/N (%). Continuous variables were tested by the non‐parametric Mann‐Whitney *U* test, and categorical variables were tested by a corrected formula of χ^2^ test. Correlation analysis was performed using Spearman rank correlation. A logistic regression model was established when analysing the independent influencing factors of visual acuity prognosis in LHON patients treated with rAAV2‐ND4 gene therapy. Significant indices in univariate analysis and indices suspected to exhibit strong correlations with prognosis were included. Independent correlation factors of visual acuity prognosis after gene therapy were screened by the “forward: LR” test, with *P* < 0.05 indicating statistically significant differences.

## 3 RESULTS

### 3.1 Patient characteristics at baseline

Among the 53 patients (53 eyes), 48 (90.57%) were male and five (9.43%) were female. The mean age at onset was 16.17 ± 4.94 years (range 5‐27 years). The mean duration of disease was 37.25 ± 48.12 months (range: 2‐312 months). The mean baseline VFI value of the injected eye was 20.77 ± 23.06% (range: 0‐84%). The mean baseline MD value of the injected eye was −26.13 ± 7.67 dB (range: −6.85‐35.52 dB). The mean baseline BCVA was 1.84 ± 0.38 logMAR (range: 0.7‐2.6 logMAR).

### 3.2 Univariate analysis

Among the 53 LHON patients (53 eyes) in this study, 24 (45.3%) and 28 (52.8%) injected eyes were included in the BCVA improvement group, while 29 (54.7%) and 25 (47.2%) were included in the non‐improvement group at 1 and 3 months, respectively, after the gene therapy. Correlations were analysed between baseline characteristics and BCVA changes of the injected eye at 1 and 3 months after the administration of gene therapy; the results of the univariate analysis are presented in Table 1. There were no statistically significant differences in sex, age at onset, duration of disease, VFI, MD or baseline BCVA between the injected eyes of the BCVA improvement and non‐improvement groups at 1 month. However, there were statistically significant differences in VFI and MD values between the injected eye of the BCVA improvement and non‐improvement groups at 3 months after the administration of gene therapy (*P* = 0.028, 0.026); the BCVA improvement group showed greater VFI and smaller absolute MD values, compared with those of the non‐improvement group. Notably, there were no statistically significant differences in sex, age at onset, disease course or baseline BCVA between the two groups. The correlation between VFI and MD was statistically significant (*P* < 0.001; correlation coefficient *r* = 0.910).

**Table 1:** Correlations between baseline characteristics and BCVA changes of the injected eye at 1 and 3 months after gene therapy

Variable	Changes in BCVA at 1 month		*P*	Changes in BCVA at 3 months		*P*
	Improvement	Non‐improvement		Improvement	Non‐improvement	
Sex (M/F)	22/2	26/3	1.000	27/1	21/4	0.283
Age at onset (years)	16.04 ± 4.31	16.28 ± 5.48	0.795	16.36 ± 4.62	15.96 ± 5.36	0.648
Duration of disease (months)	29.33 ± 26.86	43.79 ± 60.09	0.271	33.54 ± 29.15	41.40 ± 63.47	0.993
VFI (%)	29.21 ± 27.63	13.79 ± 15.79	0.053	27.68 ± 26.02	13.04 ± 16.51	0.028
MD (dB)	−23.81 ± 9.21	−28.05 ± 5.59	0.136	−23.85 ± 8.53	−28.68 ± 5.73	0.026
Baseline BCVA (logMAR)	1.94 ± 0.31	1.76 ± 0.42	0.164	1.94 ± 0.27	1.73 ± 0.46	0.099

Abbreviations: BCVA, best‐corrected visual acuity; MD, mean deviation; VFI, visual field index.

### 3.3 Logistic regression analysis

VFI, MD and baseline BCVA were included in the logistic regression model to assess factors independently correlated with visual acuity prognosis in LHON patients after the administration of gene therapy (1 and 3 months) using the “forward: LR” test. Logistic regression showed statistically significant differences in baseline VFI and BCVA, indicating that they were factors independently correlated with visual acuity prognosis (Table 2).

**Table 2:** Logistic regression analysis of factors independently correlated with injected eye BCVA changes in Leber's hereditary optic neuropathy patients at 1 and 3 months after gene therapy

Independent factor	1 month			3 months		
	Regression coefficient (B)	*P*	Exp(B)	Regression coefficient (B)	*P*	Exp(B)
VFI	0.056	0.007	1.058	0.064	0.008	1.066
Baseline BCVA	2.931	0.016	18.743	3.365	0.009	28.942

Abbreviations: BCVA, best‐corrected visual acuity; VFI, visual field index.

## 4 DISCUSSION

Since our team completed the world's first case of gene therapy for LHON in 2011, gene therapy was completed for 9 LHON patients in the first clinical trial; six patients showed significantly improved visual acuity, achieving an effective rate of 66%.^10^ Among the 53 patients receiving gene therapy in the current study, 24 and 28 patients showed improvement in visual acuity; 29 and 25 patients did not show improvements in visual acuity at the 1‐ and 3‐month follow‐up visits, respectively. Gene therapy had good outcome in some patients, and a poor outcome in others. Such differences in visual acuity after treatment have led us to further explore factors that influence the prognosis of gene therapy, in order to help guide future treatment.

Before the advent of gene therapy, there was no treatment for LHON that exhibited confirmed efficacy. Spontaneous visual recovery was observed in 4% to 25% of the patients with mutations at site 11 778.^2^, ^9^, ^14^, ^15^, ^16^, ^17^ Young age at onset,^18^ slow disease progression,^19^ mild degree of vision loss^3^ and thicker retinal nerve fibre layer^20^ were considered predictors of good visual acuity recovery. Analysis of the factors influencing spontaneous visual acuity recovery showed that baseline characteristics may also affect the visual acuity prognosis of LHON patients with mutations at site 11 778 who were treated with gene therapy.

Fifty‐three LHON patients (53 eyes) with mutations at site 11 778 were treated with gene therapy in this study. Univariate analysis showed statistically significant differences in VFI and MD values between the two groups at 3 months after treatment (*P* = 0.028, 0.026). Moreover, a study by Mashima et al 3 demonstrated that patients with better baseline visual acuity are more likely to exhibit spontaneous visual acuity recovery. Therefore, baseline VFI, MD and BCVA were included in the logistic regression model for analysis of independent correlation factors for visual acuity prognosis; the results at both 1 and 3 months after treatment showed statistically significant differences in baseline VFI and BCVA (*P* = 0.007 and 0.016 at 1 month, and *P* = 0.008 and 0.009 at 3 months, respectively), indicating that evaluation of the stability and reliability of baseline VFI and BCVA can aid in predicting visual acuity outcome in LHON patients treated with gene therapy, and that VFI and MD have a strong correlation (Spearman correlation coefficient 0.910). Therefore, MD may also be correlated with the prognosis of gene therapy. Because retinal ganglion cells (RGCs) are the structural basis for the recovery of optic nerve function, the gene therapy approach is to transfect normal, non‐mutated ND4 into RGCs at the lesion site of the patient, in order to substitute the physiological function of mutated ND4 in the patient, thus providing treatment for LHON.^8^ LHON patients with better basal visual function have more functional RGCs, and more normal ND4 protein is expressed by the gene medicine reaching the target cells. Both BCVA and visual field parameters are indicators for assessing visual function; thus, baseline VFI and BCVA were likely to be positively correlated with the visual acuity prognosis of gene therapy.

Duration of disease did not lead to a statistically significant difference in visual acuity changes in LHON patients treated with rAAV2‐ND4 gene therapy (*P* = 0.271 at 1 month and *P* = 0.993 at 3 months). In a female patient with the longest duration of disease (26 years), visual acuity improved from 1.0 logMAR at baseline to 0.7 logMAR at 3 months after treatment; in a male patient with the shortest duration of disease (2 months), visual acuity improved from 1.7 logMAR at baseline to 1.3 logMAR, both showing significant improvement of visual acuity. This indicates that duration of disease is not a factor influencing the efficacy of gene therapy. This also suggests that the function of RGCs cannot be completely lost over time, which may be related to Neil Howell's hypothesis regarding incomplete degeneration or reversible loss of functional RGCs.^21^

Previous studies have demonstrated that one of the prognostic factors correlated with spontaneous visual acuity recovery is age at onset: younger age at onset is associated with greater likelihood of visual acuity recovery.^18^, ^22^, ^23^, ^24^, ^25^ However, some studies have shown that visual acuity prognosis is also poor in patients with young age at onset.^26^ The present study demonstrated that age at onset is not correlated with visual acuity prognosis in LHON patients treated with gene therapy. In contrast to spontaneous visual acuity recovery, upon intervention with gene therapy, we caused the mitochondria to express normal ND4 protein, thereby restoring RGC function and improving visual function. Notably, the recovery mechanism is unclear in patients who exhibit spontaneous visual acuity recovery and may dramatically differ from that of gene therapy.

In this study, we analysed the baseline data and primary eye examination parameters in 53 LHON patients with mutation at site 11 778. We found that baseline VFI and BCVA are effective indicators for evaluating the prognosis of gene therapy, with greater VFI and better BCVA at baseline predicting better outcome of gene therapy. There is a strong correlation between VFI and MD; MD may also be correlated with the prognosis of gene therapy. This conclusion provides a basis for predicting the efficacy of gene therapy in LHON patients with mutation at site 11 778 and may guide subsequent treatment. Our team will continue the long‐term follow‐up of these patients treated with gene therapy to further explore other factors that may affect the efficacy of gene therapy.

## CONFLICT OF INTEREST

None declared.
